# Audit on COVID-19 Vaccine Uptake and Hesitancy Amongst Pregnant or Postnatal Patients Under the Care of a Perinatal Mental Health Community Team

**DOI:** 10.1192/bjo.2022.471

**Published:** 2022-06-20

**Authors:** Bosky Nair, Alex Oh, Mahdi Alsahaf, Unwana Etteh

**Affiliations:** Kent and Medway NHS and Social Care Partnership Trust, Maidstone, United Kingdom

## Abstract

**Aims:**

Women with perinatal mental illness are at increased risk for severe illness with COVID-19. Vaccination against COVID-19 is strongly recommended by JCVI (Joint Committee on Vaccination and Immunisation) and RCOG guidance. Mental health professionals should proactively inform their patients about COVID-19 vaccination and also address any concerns or misinformation, should they be raised. The aim of this audit was to evaluate the rate of uptake of the COVID-19 vaccine among patients under the West Kent community perinatal mental health team. In addition, we aimed to identify factors that deter patients from taking the COVID-19 vaccine. In patients who were hesitant to take the vaccine, we offered further information to aid their decision-making process.

**Methods:**

We identified patients under the care of the West Kent perinatal mental health community team on 27/10/2021. We excluded patients who were discharged from the team in subsequent weeks during data collection. We collected patient demographics including highest level of education, ethnicity, religion and socio-economic status. Patients’ COVID-19 vaccine status was obtained via GP records or through telephone contact.

If patients had not had their COVID-19 vaccine, they were contacted to enquire whether they were planning to take the vaccine, if not, to ascertain reasons for refusal and whether they wanted additional information about the vaccine. Those women who requested additional information were offered the RCOG information sheet and decision aid.

**Results:**

Amongst 86 patients included in the audit, 59% (n = 51) had taken both dose of the COVID-19 vaccine and 12% (n = 10) had taken a single dose. 29% (n = 25) were unvaccinated.

68% (n = 17) of unvaccinated patients were pregnant and 32% (n = 8) were postnatal. All women who did not accept COVID-19 vaccine were contacted to offer further information. Following this contact, 39% (n = 9) decided to accept the vaccine, 52% (n = 12) refused the vaccine and 26% (n = 6) were uncertain but were willing to consider taking the vaccine in the future.

The reasons for hesitancy in accepting the vaccine included a lack of trust in the vaccine, concerns around its development over a short period of time, concerns around close associates experiencing illness or side effects after taking the vaccine and scepticism over efficacy of the vaccine. Few women did not wish to take the vaccine during their pregnancy, but were willing to consider it after the birth of their baby.

**Conclusion:**

We identified potential areas to optimise uptake of COVID-19 vaccines by discussing the importance, safety, efficacy and providing up-to-date information regarding COVID-19 vaccine in the perinatal period.

